# A new species of the carpenter bee genus *Xylocopa* from the Sarawat Mountains in southwestern Saudi Arabia (Hymenoptera, Apidae)

**DOI:** 10.3897/zookeys.716.21150

**Published:** 2017-11-23

**Authors:** Michael S. Engel, Abdulaziz S. Alqarni, Mohamed A. Shebl, Javaid Iqbal, Ismael A. Hinojosa-Díaz

**Affiliations:** 1 Division of Entomology, Natural History Museum, and Department of Ecology & Evolutionary Biology, 1501 Crestline Drive – Suite 140, University of Kansas, Lawrence, Kansas 66045-4415, USA; 2 Division of Invertebrate Zoology, American Museum of Natural History, Central Park West at 79th Street, New York, New York 10024-5192, USA; 3 Department of Plant Protection, College of Food and Agriculture Sciences, King Saud University, P.O. Box 2460, Riyadh 11451, Kingdom of Saudi Arabia; 4 Department of Plant Protection, Faculty of Agriculture, Suez Canal University, Ismailia 41522, Egypt; 5 Department of Entomology, Muhammad Nawaz Shareef University of Agriculture, Multan, Pakistan; 6 Departamento de Zoología, Instituto de Biología, Universidad Nacional Autónoma de México, Mexico City, DF, Mexico

**Keywords:** Apoidea, Anthophila, Xylocopini, Arabian Peninsula, taxonomy, carpenter bees

## Abstract

A new species of the carpenter bee genus *Xylocopa* Latreille (Xylocopinae: Xylocopini) is described and figured from two localities in southern Saudi Arabia. Xylocopa (Koptortosoma) sarawatica Engel, **sp. n.** is a relatively small species similar to the widespread *X. pubescens* Spinola, but differs in the extent of maculation in males, setal coloration of both sexes, and male terminalia. A revised key to the species of *Xylocopa* in Saudi Arabia is provided.

## Introduction

The genus *Xylocopa* Latreille (Xylocopinae: Xylocopini) comprises approximately 375 species of large, robust bees, superficially resembling bumble bees (Apinae: Bombini), that are distributed throughout the world (Michener 2007). Species are commonly dubbed ‘large carpenter bees’ as most species, like their relatives among the small carpenter bees (genus *Ceratina* Latreille), typically nest in dead wood, stems, or similar cavities (Michener 2007). During recent collecting in southern Saudi Arabia we have encountered small individuals of *Xylocopa* superficially resembling the larger and more widespread Xylocopa (Koptortosoma) pubescens Spinola (treated in an earlier work as *X. aestuans* (Linnaeus) (Hannan et al. 2012), a species with which it has been comingled: e.g., Lieftinck 1964). Herein we provide a formal description of this species in the hopes that it might be sought from additional localities and its nests discovered. *Xylocopa pubescens* and X. (Ctenoxylocopa) sulcatipes Maa nest frequently in *Calotropis procera* (Aiton) (Asclepiadaceae) or *Phoenix datylifera* L. (Arecaeae) elsewhere in Saudi Arabia (Hannan et al. 2012), and the former was found nesting in *C. procera* at sites near the type locality for the species described herein (Engel pers. obs.). It is hoped that continued hunting for the new species in the Al-Baha or ‘Asir Regions might eventually recover nests and immature stages of this smaller Arabian *Xylocopa*.

## Materials and methods

Material is deposited in the King Saud University Museum of Arthropods, Plant Protection Department, College of Food and Agriculture Sciences, King Saud University, Riyadh, Kingdom of Saudi Arabia (**KSMA**), and the Division of Entomology (Snow Entomological Collections), University of Kansas Natural History Museum, Lawrence, Kansas, USA (**SEMC**). Morphological terminology in the description and key is based on that of Engel (2001) and Michener (2007). Photomicrographs were prepared with a Canon 7D digital camera attached to an Infinity K-2 long-distance microscope lens and illuminated by a Xenon flash system. Measurements were taken with an ocular micrometer on an Olympus SZX12 stereomicroscope. The formats for the diagnosis and description follows those used elsewhere in the systematics of African-Asiatic *Xylocopa* (e.g., Eardley 1983; Hannan et al. 2012), and these data are presented to enhance our current circumscriptions of species of *Koptortosoma* (e.g., Engel 2011, Gonzalez et al. 2013), provide basic information from which broader synthetic patterns may eventually be established (Grimaldi and Engel 2007), and build a richer understanding of the Saudi bee fauna, particularly of the Al-Baha Region (El-Hawagry et al. 2013).

## Systematics

### Genus *Xylocopa* Latreille

#### Subgenus Koptortosoma Gribodo

##### Xylocopa (Koptortosoma) sarawatica

Taxon classificationAnimaliaHymenopteraApidae

Engel sp. n.

http://zoobank.org/E5684338-7F59-4100-952B-641B9E1A37E7

Figs 1–3 , 4–7 , 8–10

###### Diagnosis.

The new species most closely resembles the more widespread *X. pubescens*, albeit at about three-quarters of the body size or less (*vide* Key, *infra*). Aside from size, *X. sarawatica* can be most easily distinguished in females by the entirely black or dark fuscous facial setae (versus white to off-white setae intermixed with black setae in *X. pubescens*) and the presence of yellow setae on the metanotum (entirely black to dark fuscous in *X. pubescens*). Males can be recognized by the presence of yellow maculation along the apical margin of the clypeus and on the procoxal spine (absent in *X. pubescens*), the entirely black or dark fuscous setae of the basitarsi (some white to yellow setae present in *X. pubescens*), and in the form of the male terminalia (*cf.* Figs 4–7 with those from Hannan et al. 2012: their figures 7–11, listed as *X. aestuans* but actually *X. pubescens*). In particular, the paramedial lobes of the eighth metasomal sternum are more widely spaced and broader, much broader than the spiculum (Fig. 4) (together scarcely broader than spiculum in *X. pubescens*: fig. 11 in Hannan et al. 2012), the genital capsule is broader anteriorly (Figs 6, 7) (anteriorly narrowed in *X. pubescens*: figs 7, 9 in Hannan et al. 2012), the medial dorsal margins of the gonocoxae meet along approximately the basal one-half of length before diverging to the broad mediodorsal lobes (Fig. 7) (margins meet along approximately basal one-quarter to one-third of length in *X. pubescens*: fig. 7 in Hannan et al. 2012); the gonostylar apex is much broader (Figs 6, 7) (more slender in *X. pubescens*: figs 7, 9 in Hannan et al. 2012); and the penis valves are widened apically at point of arching ventrally before tapering to acute apex (Fig. 6) (penis valves uniformly slender along apical portion of length at same point in *X. pubescens*: fig. 9 in Hannan et al. 2012).

**Figures 1–3.**
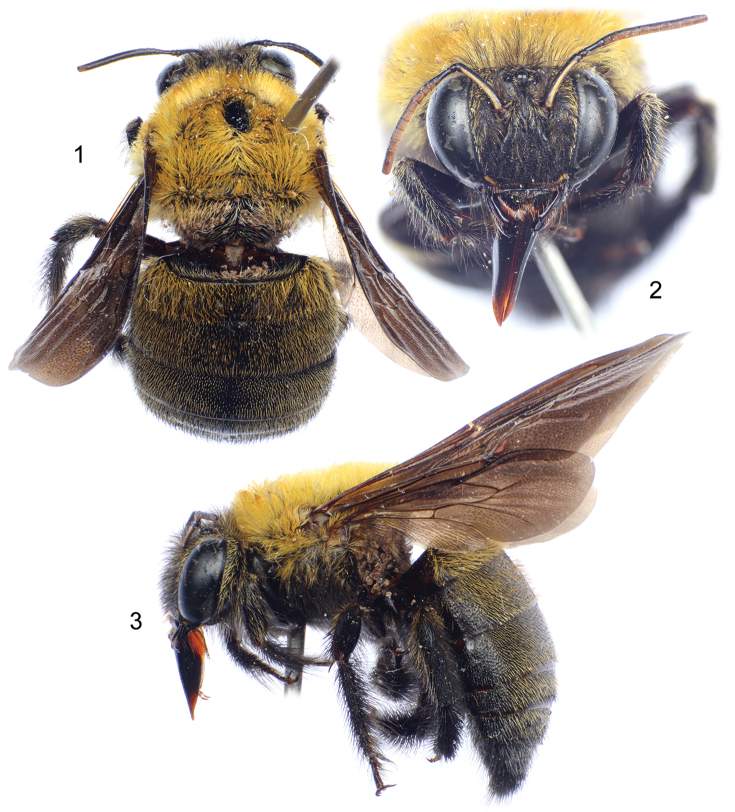
Male of Xylocopa (Koptortosoma) sarawatica Engel, sp. n., from southern Saudi Arabia. **1** Dorsal habitus **2** Facial view **3** Lateral habitus.

###### Description.

**Figures 4–7.**
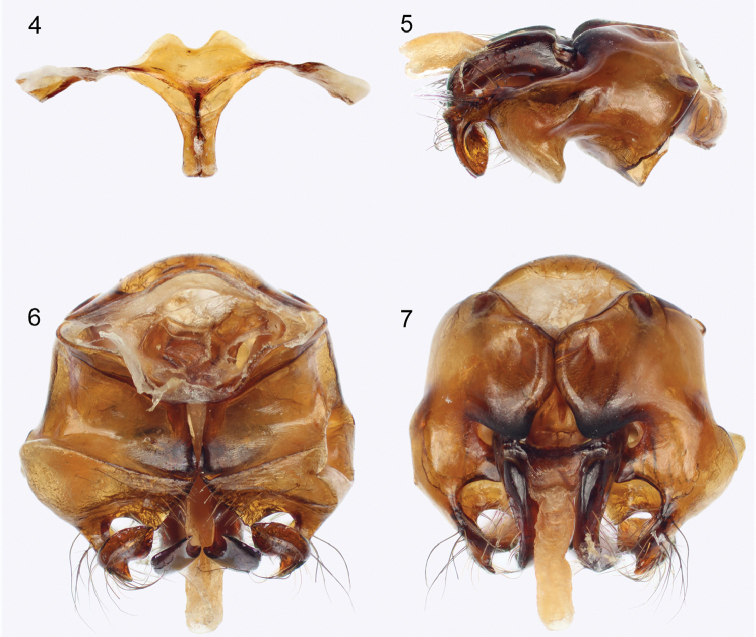
Male terminalia of Xylocopa (Koptortosoma) sarawatica Engel, sp. n. **4** Metasomal sterna VII+VIII **5** Genital capsule in profile **6** Genital capsule in ventral view **7** Genital capsule in dorsal view.

♂: Total body length 14.9 mm; forewing length (excluding tegula) 11.7 mm. Head broader than long; head length (vertex to clypeal apical margin in facial view) 2.7 mm, maximum width 4.2 mm. Compound eyes slightly more convergent below than above, with inner orbit weakly concave; inner orbital margin well separated from antennal torulus; upper interorbital distance 2.2 mm, lower interorbital distance 1.9 mm, maximum interorbital distance 2.4 mm; compound eye length 2.5 mm. Ocelli situated high on face, posterior tangent of ocelli at about upper orbital tangent; ocellocular distance approximately twice diameter of median ocellus; interocellar distance approximately 2.5 times diameter of median ocellus; ocelloccipital distance approximately 1.5 times diameter of median ocellus. Scape long, length 1.3 mm, exceeding upper compound eye tangent; first flagellomere elongate, longer than combined lengths of second and third flagellomeres; antennal toruli separated from clypeus by slightly less than torular diameter. Clypeus flat, without median ridge or line, longer than frons, with dorsolateral margins raised above bordering paraocular areas; paraocular areas without sulci, carinae, or depressions. Labrum transverse, with apical margin medially concave, surface with rounded, transverse ridge at midlength and short mediolongitudinal ridge from base to transverse ridge. Mandible bidentate, without internal tooth; malar area linear. Intertegular distance 4.2 mm; apical margin of mesoscutellum sharply angled, sharp angle separating dorsal surface from obliquely ventral subvertical surface, in profile projecting over metanotum and strongly declivitous propodeum as a short, thin flange; metanotum subhorizontal; propodeum entirely declivitous, triangular area of propodeum absent. Forewing with basal vein confluent with 1cu-a; three submarginal cells; 1Rs+M with minute veinal stub extending into first submarginal cell at about midlength; 2Rs elongate, apically arched, giving second submarginal cell an elongate posterobasal extension; 1rs-m comparatively straight, slightly distad 1m-cu; 2rs-m broadly arched, greatly distad 2m-cu; 2m-cu entering third submarginal cell at approximately apical third of cell length. Procoxal spine short; apex of metabasitibial plate acute, situated slightly before metatibial midlength, its anterior margin short and posterior border extending as a carina for some distance basally. Metasoma with dorsal-facing and anterior-facing surfaces of tergum I abruptly and with an angular separation; vertical fold of tergum I with foveate depression; apical margin of sternum I entire and medially pointed (not emarginate or concave); sternum V with broad, medial lobe along apical margin; male hidden sterna and genital capsule as in Figures 4–7.

**Figures 8–10.**
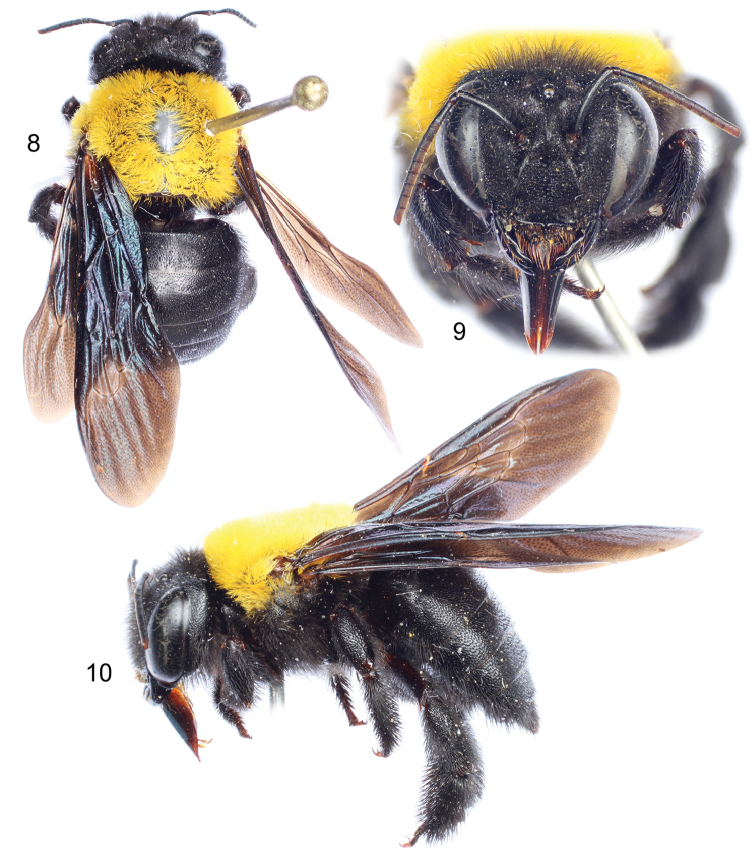
Female of Xylocopa (Koptortosoma) sarawatica Engel, sp. n. **8** Dorsal habitus **9** Facial view **10** Lateral habitus.

Integument black or dark brown throughout without metallic highlights, except yellow on following surfaces: small spot at extreme base of mandible, four narrow streaks along apical margin of clypeus, underside of scape along its length, and procoxal spine. Ventral surface of flagellum, excluding first flagellomere, lighter reddish brown; mouthparts dark brown to black; apical tarsomeres and pretarsal claws reddish brown. Wing membranes infuscate and apically papillate, with exceedingly faint violaceous highlights anteriorly; veins dark brown.

Clypeus with coarse, small punctures separated by less than a puncture width, slightly more widely spaced in small area centrally, integument between dull and microscopically imbricate, without impunctate medial line or ridge; punctures of face as on clypeus except largely contiguous, impunctate around frontal line and ocelli; ocellocular area with punctures separated by less than a puncture width, integument between dull and imbricate; punctures posterior to ocelli similar to those of ocellocular area; vertex with punctures separated by less than a puncture width; punctures of gena largely separated by less than a puncture except in some areas separated by up to a puncture width, integument between punctures imbricate. Mesoscutum and mesoscutellum with small, nearly contiguous punctures at base of setae, impunctate on central disc of mesoscutum, surface polished, integument between setigerous punctures apparently faintly imbricate; tegula largely impunctate and shining; punctures of pleura small and coarse, separated by a puncture width or less, becoming slightly more widely spaced ventrally; metanotum imbricate and largely impunctate except faint punctures at bases of setae; propodeum with setigerous punctures separated by a puncture width or slightly more, integument between punctures imbricate. Metasomal tergum I with small punctures separated by a puncture width or more, integument between punctures dull and imbricate, punctures becoming progressively more densely packed laterally; punctures centrally on terga II–III similar to those on tergum I except slightly more closely spaced, particularly so apically on tergum III; punctures centrally on terga IV–V less well defined and denser than those on discs of preceding terga; punctures centrally on tergum VI similar to preceding terga except even more poorly defined and shallow; sterna with setigerous punctures widely spaced in basal halves, becoming more closely spaced apically and laterally on each sternum.

Pubescence generally yellow on face and upper surfaces, intermingled with dark fuscous to black setae and some areas of white as noted; dark fuscous to black setae dominant on lower surfaces. Face with darker, branched setae erect to suberect dominant on clypeus, lower paraocular areas, and lower face, intermingled with long, branched, erect to suberect yellow setae, such yellow setae becoming more numerous in upper half of face, with short, branched, appressed to subappressed setae present along upper ocular borders, ocellocular area, vertex, and gena; vertex with erect, simple, black setae intermingled among other setal types; gena with long, yellow setae similar to those on face dominating, blending to longer, dark fuscous to black setae ventrally and on postgena. Setae on mesosomal dorsum, including tegula, long, branched, yellow, and densely covering integument except centrally on disc of mesoscutum and on undersurface of projecting mesoscutellum; setae on upper portion of pleura and upper border of propodeal lateral surface yellow, blending ventrally to fuscous setae, all such setae erect, long, and branched; setae on posterior surface of propodeum largely, erect, long, branched, and white, more sparsely distributed than on pleura. Legs with predominantly black setae except intermixed with yellow setae on outer anterior surfaces of tibiae, particularly basally; posterior setae of pro- and mesotibiae and pro- and mesobasitarsi extremely elongate, those of basitarsi nearly as long as basitarsi; metabasitarsus with greatly elongate setae albeit distributed more regularly on anterior and posterior surfaces. Metasomal terga I and II with predominantly moderately long, suberect to erect, yellow setae with few or no branches, such setae becoming white at extreme lateral margins except on tergum II where such white and yellow setae are intermixed with some long, suberect, black setae, particularly posterolaterally; long yellow setae of tergum II gradually sparser toward apex and replaced by shorter, subappressed, stouter, simple, yellow setae; terga III–V with such short, appressed to subappressed, stout, simple, yellow setae predominant except laterally with long, black or white, simple, setae; tergum VI with short, subappressed, yellow setae dominant over basal half then gradually replaced by longer, suberect, white or black, setae in apical half, black setae particularly elongate apicolaterally; sterna with largely suberect, simple, white setae; sternum IV with white setae replaced apically by subappressed to suberect, simple, black setae; sternum V with black, subappressed to suberect, simple setae although a few with minute branches, such setae of moderate length over disc and greatly elongate and more erect apicolaterally.

♀: Total body length 14.8–17.0 mm; forewing length (excluding tegula) 11.7–12.6 mm. Head broader than long; head length (vertex to clypeal apical margin in facial view) 3.3–3.7 mm, maximum width 4.6–5.2 mm. Compound eyes slightly more convergent below than above, with inner orbit weakly concave; inner orbital margin well separated from antennal torulus; upper interorbital distance 2.6–2.9 mm, lower interorbital distance 2.2–2.5 mm, maximum interorbital distance 2.8–3.2 mm; compound eye length 2.9–3.3 mm. Ocelli situated high on face, posterior tangent of ocelli at about upper orbital tangent; ocellocular distance approximately 2.5 times diameter of median ocellus; interocellar distance approximately 2.5 times diameter of median ocellus; ocelloccipital distance approximately twice diameter of median ocellus. Scape long, length 1.5–1.7 mm, exceeding upper compound eye tangent; first flagellomere elongate, longer than combined lengths of second and third flagellomeres; antennal toruli separated from clypeus by slightly less than torular diameter. Clypeus flat, without median ridge or line, longer than frons, with dorsolateral margins raised above bordering paraocular areas; paraocular areas without sulci, carinae, or depressions. Labrum transverse, with four blunt tubercles medially. Maxillary palpus pentamerous (as in X. pubescens, and contrary to many Koptortosoma *s.str.*). Mandible bidentate, without internal tooth; malar area linear. Intertegular distance 4.5–4.9 mm; apical margin of mesoscutellum sharply angled, sharp angle separating dorsal surface from obliquely ventral subvertical surface, in profile projecting over metanotum and strongly declivitous propodeum as a short, thin flange; metanotum subhorizontal; propodeum entirely declivitous, triangular area of propodeum absent. Apex of metabasitibial plate acute, situated slightly beyond metatibial midlength, its anterior margin short and posterior border extending as a carina for some distance basally. Metasoma with dorsal-facing and anterior-facing surfaces of tergum I abruptly and angulately separated; vertical fold of tergum I with foveate depression; terga II–VI lacking graduli; pygidial spine narrow, parallel-sided, unarmed; apical margin of sternum I entire and medially pointed (not emarginate or concave); sternal margins unmodified, straight.

Integument black or dark brown throughout, without yellow maculation or metallic highlights. Wing membranes infuscate, with exceedingly faint violaceous highlights anteriorly; veins dark brown.

Clypeus with coarse, small punctures separated by much less than a puncture width, slightly more widely spaced in small area centrally, integument between dull and microscopically imbricate, without impunctate medial line or ridge; punctures of face as on clypeus except largely contiguous, impunctate around frontal line and ocelli; ocellocular area with punctures separated by a puncture width or frequently less, integument between dull and imbricate; punctures posterior to ocelli similar to those of ocellocular area; vertex with punctures separated by less than a puncture width; punctures of gena largely separated by less than a puncture width except in some small places separated by up to a puncture width, integument between punctures imbricate. Mesoscutum and mesoscutellum with small punctures at base of setae, impunctate on central disc of mesoscutum, surface polished, integument between setigerous punctures apparently faintly imbricate; tegula largely impunctate and shining, with some punctures anteriorly; punctures of pleura small and coarse, separated by a puncture width or less, becoming slightly more widely spaced ventrally; metanotum imbricate and largely impunctate except faint punctures at bases of setae; propodeum with setigerous punctures separated by a puncture width or slightly more, integument between punctures imbricate. Metasomal tergum I with small punctures separated by a puncture width or more, integument between punctures dull and imbricate, punctures becoming progressively more densely packed laterally until nearly contiguous; punctures centrally on terga III–V more widely spaced than those of preceding terga; tergum VI with punctures of disc less well defined and denser than those on discs of preceding terga; sterna with setigerous punctures largely separated by more than a puncture width except becoming dense and somewhat smaller laterally and apically, narrow impunctate area medially on sternum II and less so on sternum III.

Pubescence generally dark fuscous to black throughout except bright yellow on mesoscutum, tegula, mesoscutellum, metanotum, and uppermost borders of pleura and propodeum. Setae of face, vertex, gena, and postgena moderately long to long, suberect to erect, and with a few branches to simple, those with branches typically somewhat shorter, setae never obscuring integument; setae on mesosomal dorsum long and with minute branches, densely covering integument except centrally on disc of mesoscutum and on undersurface of projecting mesoscutellum, and exceptionally sparse on metanotum; pleura and propodeal lateral surface with long, erect setae with many branches, such setae somewhat obscuring integument; posterior surface of propodeum with short, largely simple setae, more sparsely distributed. Legs with abundant, long setae. Metasomal terga with short setae scattered over integument, those of dorsal-facing surface of tergum I largely erect and longer than those of discs of remaining terga; remaining terga with setae largely appressed to suberect, largely simple, not obscuring integument, setae more numerous and longer to sides and on apicalmost terga; sterna with long, suberect, simple setae.

###### Holotype.

♂, Saudi Arabia, Baha [Al-Baha Region], Thee Ain [Thy ‘Ain] Village, 690 m, 19°55'59.61"N, 41°26'41.41"E, 25-v-2012 [25 May 2012], M.A. Hannan (SEMC).

###### Paratypes.

1♀, Saudi Arabia, Asir [‘Asir Region], Abha, Sodah, nr. dam, 2500 m, 18°14'11.64"N, 42°24'49.96"E, 22-v-2012 [22 May 2012], M.S. Engel (SEMC); 1♀, Saudi Arabia, Al Baha [Al-Baha Region], Thy Ein [Thy ‘Ain] village, 690 m, 19°55'59.61"N, 41°26'41.41"E, 25-v-2012 [25 May 2012], M.S. Engel (SEMC); 4♀♀, Saudi Arabia, Baha [Al-Baha Region], Thee Ain [Thy ‘Ain] Village, 690 m, 19°55'59.61"N, 41°26'41.41"E, 25-v-2012 [25 May 2012], M.A. Hannan (3♀♀ SEMC, 1♀ KSMA); 1♂, 1♀, Saudi Arabia, Al Baha [Al-Baha Region], The Ain [Thy ‘Ain], 5.5.2015 [5 May 2015], M. Shebl (KSMA); 1♀, Saudi Arabia, [Al-Baha Region], Thee Ain [Thy ‘Ain], 20 km S. of Baha, 13.X.2010 [13 October 2010], N 19°55'54", E 41°26'29", Al Dafer, H., Kondratieff, B., Fadl, H. & El Gharbawy, A. (KSMA).

###### Remarks.

As is the case with many *Xylocopa*, the male holotype has a profusion of immature mites present on the propodeum.

###### Etymology.

The specific epithet is based on the Sarawat Mountain range from which the species was collected, either at elevation or along the escarpment over the Tihāmah.

### Key to species of *Xylocopa* in Saudi Arabia (updated from Hannan et al. 2012)

**Table:** 

1	Males	**2**
–	Females	**4**
2	Body with abundant, dense, yellow pubescence throughout, particularly dorsally; first metasomal tergum with subhorizontal dorsal surface abruptly and angulately separated from declivitous anterior-facing surface; first metasomal tergum with gradulus transverse, lateral extremities not directed posteriorly	**3**
–	Body covered by largely fuscous to black pubescence except face, dorsum of mesosoma, and apicolateral patches of first metasomal tergum with predominantly white or pale setae; first metasomal tergum with subhorizontal dorsal surface rounding into declivitous anterior surface; first metasomal tergum with gradulus laterally curved posteriorly	***X.sulcatipes* Maa**
3	Clypeus without maculation, entirely black; procoxal spine black; basitarsi with white or yellow setae on outer anterior surfaces; large bees, body length over 18.5 mm, forewing length over 15 mm	***X.pubescens* Spinola**
–	Clypeus with some small yellow maculation along apical margin; procoxal spine yellow; basitarsi with setae entirely black to dark fuscous; smaller bees, body length under 15.5 mm, forewing length under 12.5 mm	***X.sarawatica* Engel, sp. n.**
4	Mesosomal dorsum densely covered by yellow pubescence, such setae obscuring integument; face with largely white or pale pubescence; pygidial plate unarmed; posterodorsal margin of mesoscutellum projecting beyond posterior margin of metanotum; mandible bidentate at apex	**5**
–	Mesosomal dorsum largely covered by black pubescence, such setae not obscuring integument; face with largely black pubescence; pygidial plate armed on each side with subapical spine; mesoscutellum not projecting over metanotum, apical margin rounded in profile; mandible tridentate at apex	***X.sulcatipes* Maa**
5	Face (including clypeus) with abundant white setae intermingled with black setae; metanotal setae entirely dark fuscous; large bees, body length over 22 mm, forewing length over 16.5 mm	***X.pubescens* Spinola**
–	Face (including clypeus) with setae entirely dark fuscous to black; metanotal setae yellow; smaller bees, body length under 17.5 mm, forewing length under 13 mm	***X.sarawatica* Engel, sp. n.**

## Discussion

The new species from Saudi Arabia belongs to the diverse and widespread subgenus Koptortosoma Gribodo, a group that was once split into several different subgenera (e.g., Hurd and Moure 1963). Over the last half of the 20^th^ century, these other subgeneric units were gradually synonymized with *Koptortosoma*. In a study of Central Asiatic Xylocopini, Maa (1954) united *Koptortosoma* with *Maiella* Michener (followed by Hurd 1959), while following a cladistic analysis of the tribe Xylocopini, Minckley (1998) united *Cyaneoderes* Ashmead, *Afroxylocopa* Hurd & Moure, *Oxyxylocopa* Hurd & Moure, and *Cyphoxylocopa* Hurd & Moure. Lastly, Michener (2000) added *Lieftinckella* Hurd & Moure to this list of synonyms, arriving at our modern concept of the subgenus. As currently constituted, *Koptortosoma* encompasses approximately 150 species ranging from sub-Saharan Africa to Europe, across Asia into Australasia (Michener 2007). However, it is increasingly apparent that *Koptortosoma* as so defined is not monophyletic (e.g., Leys et al. 2000, 2002, Kawazoe et al. 2008). Indeed, *Koptortosoma* perhaps represents one of the greatest challenges in the systematics of the large carpenter bees, and its species should likely be relegated again into three or four subgenera, with *Koptortosoma* *s.str.* (including *Afroxylocopa* and *Oxyxylocopa*) applicable for those African species. For the Australasian, Indomalayan, and other Asiatic groups, it may prove worthwhile to resurrect the names *Cyaneoderes*, *Maiella* (including *Cyphoxylocopa*), and *Lieftinckella* (best united with *Alloxylocopa*). Future cladistic analyses should strive to expand sampling of African and Asiatic species of *Koptortosoma* as currently defined in order to ascertain the proper boundaries of its constituent subunits.

**Figures 11–13.**
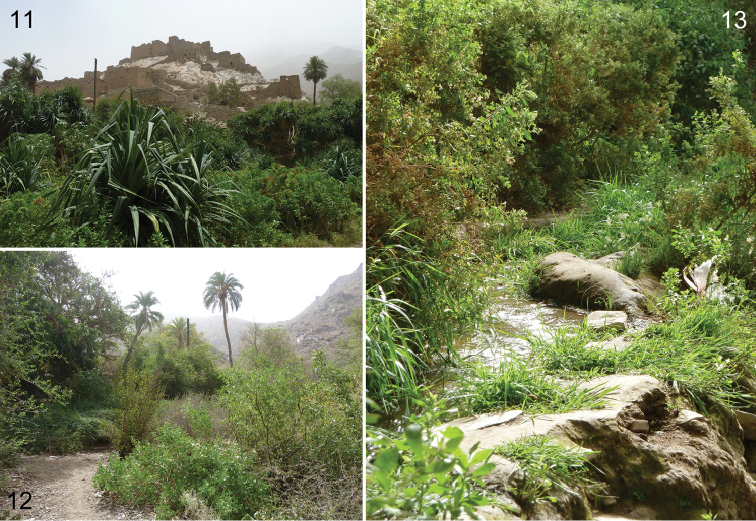
Habitat at the Thy ‘Ain type locality, near Al-Baha in Al-Baha Region, Saudi Arabia (photographs by M.S. Engel, May 2012). **11** The ancient village (the ‘Marble Village’) and surrounding vegetation **12** General vegetation at locality **13** Vegetation alongside part of small water seep.

The locality from which most of the available specimens were captured is an often-visited historical site, ‘Thy ‘Ain’ (the so-named, ‘Marble Village’: Fig. 11), along the steep road leading from the city of Al-Baha at the top of the Sarawat escarpment down to the Tihāmah. The fauna is largely Afrotropical in composition but intermingles Palearctic and Oriental elements (El-Hawagry et al. 2013), and has a rather lush desert vegetation (Figs 12, 13). Both of Arabia’s natively occurring honey bees, Apis (Apis) mellifera L. and A. (Micrapis) florea Fabricius, are common at the locality, along with various halictines, anthidiines, apine genera such as *Amegilla* Friese and *Thyreus* Panzer, as well as the xylocopines Ceratina (Pithitis) tarsata Morawitz, *Braunsapis alqarnii* Engel & Michener, and X. (C.) sulcatipes (Engel et al. 2014, Engel unpubl. data). Under the restricted subgeneric circumscriptions of Hurd and Moure (1963), *X. sarawatica* would fall among the typical African species of *Koptortosoma*, thereby further supporting the null hypothesis of a predominantly Afrotropical influence on the faunal composition of the region (El-Hawagry et al. 2013).

## Supplementary Material

XML Treatment for Xylocopa (Koptortosoma) sarawatica
